# Functional Exhaustion Limits CAR-NK Therapy in Glioblastoma but Can Be Mitigated by Early Repeated Dosing

**DOI:** 10.21203/rs.3.rs-10808000/v1

**Published:** 2026-10-04

**Authors:** Kajal Chaudhry, Nethaji Muniraj, Haili Lang, Ashley Geiger, Gage Paul, Ehsan Dowlati, Danielle Sohai, Joshua Terao, Sridevi Yadavilli, Carlos Sanchez, Eric Yvon, Christopher Lazaraski, Eugene Hwang, Matthias Holdhoff, Barbara Savoldo, Conrad Russell Cruz, Catherine Bollard

**Affiliations:** Children’s National; Children’s National; Children’s National; Children’s National; Children’s National; Georgetown University Medical Center; Children’s National; Children’s National; Children’s National; Children’s National; Children’s National; Children’s National; Children’s National; Johns Hopkins University; University of North Carolina at Chapel Hill; Children’s National; Children’s National

## Abstract

With the commercial success of Chimeric Antigen Receptor T (CAR-T) cell therapies in the blood cancer setting, there are increasing numbers of clinical trials evaluating this treatment modality in patients with solid tumors, including brain tumors. Allogeneic CAR-natural killer (NK) cell therapy offers an attractive “off-the-shelf” option using the CAR platform. However, this approach remains comparatively untested clinically, with only one published clinical trial to date in patients with central nervous system (CNS) tumors. Building on our prior in vitro characterization of allogeneic B7H3-CAR-NK for brain tumors, we compared B7H3/CD28z CAR-NK and CAR-T cells in an aggressive adult Glioblastoma Multiforme (GBM) model in vitro and in an orthotopic murine xenograft system. CAR-NK cells killed GBM targets more rapidly than CAR-T cells in vitro, and secreted higher levels of IFN-γ. However, in vivo, CAR-T cells controlled tumor growth and prolonged survival while CAR-NK cells failed to restrict tumor outgrowth despite confirmed tumor infiltration and persistence. Neither B7H3 antigen upregulation on NK cells nor trogocytosis-driven fratricide accounted for this discrepancy. Instead, we discovered that CAR-NK cells lost cytolytic activity, proliferative capacity, and cytokine secretion after multiple rounds of tumor challenge. This coincided with a downregulation of activating receptors and upregulation of inhibitory receptors. In contrast, CAR-T cells retained their cytolytic activity through four rounds of tumor challenge. While negating the consequences of tumor-induced suppression (by abrogating TGF-β signaling and increasing IL15 signaling), restored in vitro serial killing capacity and reduced in vivo tumor burden, these engineering strategies were insufficient to enhance overall survival. In contrast, administering multiple doses of NK cell products early did confer a survival benefit in preclinical models, suggesting that the CAR-NK cell platform may be more effective when utilized clinically in the adjuvant setting for patients with high-risk brain tumors such as GBM.

## INTRODUCTION

Glioblastoma multiforme (GBM) is a fast-growing, highly aggressive Grade 4 astrocytoma that originates in the brain. Patients with relapsed/refractory brain cancer have a poor prognosis and novel therapies are urgently needed. Immunotherapies based on chimeric antigen receptor T (CAR-T) and NK (CAR-NK) cells are emerging for the treatment of GBM. Several CAR-T therapies targeting single tumor-specific antigens, like IL13Rα2 (NCT02208362), HER2 (NCT01109095), and GD2 (NCT04196413) [1–3], and dual-targeting CAR-T directed against EGFRvIII and IL-13Rα2 (NCT05168423) [4], and EGFRvII and EGFR (NCT05660369) have entered early-phase clinical trials, with some early evidence of success.

Allogeneic CAR-NK platforms are also being explored clinically [5, 6] and offer an alternative “off the shelf” approach to autologous CAR-T cell therapies. While this approach has shown some success for the treatment of B-cell malignancies, clinical validation of CAR-NK therapy for solid tumors, such as GBM, is limited, with only one published clinical trial (by Burger et al. [7]) In that study, HER2-targeted CAR-NK cell therapy was evaluated in patients with recurrent glioblastoma (achieving median progression-free and overall survivals of 7 and 31 weeks, respectively). Given this limited patient data, it remains unclear if CAR-NK cells show similar potency in vivo to CAR-T in the CNS tumor setting.[8] Therefore, we assert that since the clinical experience with CAR-NK therapy lags behind CAR-T therapy in the brain tumor setting, there are significant knowledge gaps remaining which require additional preclinical work.

Several preclinical studies have assessed the challenges of CAR-NK cells in brain tumor models, including GBM.[9, 10] From these studies, we have learned that CAR-NK therapy faces numerous hurdles, including limited cell persistence, in vivo expansion, tumor trafficking, and efficacy in the immunosuppressive tumor microenvironment (TME), as well as the potential to elicit allogeneic rejection.

We have previously reported the development of B7H3-targeted CAR-NK therapy in an *in vitro* GBM model.[11] In that study, we evaluated the CAR-NK cell product against the adult GBM tumor cell lines U87 MG and A172, and the pediatric tumor lines GBM511 and GBM110. Here, we investigate the mechanistic reasons limiting CAR-NK in an *in vitro* and *in vivo* setting, using the aggressive adult GBM brain tumor U87 model, with an aim to address the challenges of translating CAR-NK therapy to the clinic for the treatment of patients with GBM.

In this current study, we find that CAR-T cells possess greater serial killing capacity in a repetitive tumor-killing model and show attenuated senescence compared to CAR-NK cells. In preclinical models, repeated antigen engagement and tumor immune suppression caused a loss of CAR-NK cell cytolytic function via multiple mechanisms, including a loss of activating receptors, increased expression of inhibitory receptors, and decreased cytokine secretion. Our studies further show that a dominant-negative receptor (DNR) can correct TGF-β-mediated NK cell dysfunction. Likewise, NK cell senescence driven by repetitive antigen engagement can be overcome by incorporating an IL-15-secreting strategy. But neither approach yielded improved survival outcomes. Thus, we sought to use an alternative strategy in this aggressive adult GBM U87 model. We demonstrate here that successful treatment with CAR-NK cells can be achieved by frequent NK cell delivery at early time points, which prevents or reduces exhaustion-driven dysfunction and sustains NK cell activity despite chronic stimulation. Finally, we propose that NK cell donor KIR/HLA considerations may have a greater impact on overall outcomes than gene modification with a CAR or DNR.

## RESULTS

### B7H3/CD28z CAR-NK cells and T cells both show efficient antitumor responses against B7H3 ^+^ targets in vitro

B7H3/CD28z CAR-engineered T cells have been successfully tested in preclinical models of CNS tumors [12, 13]. Moreover, as we reported earlier, B7H3 CAR-NK cells demonstrated greater *in vitro* cytotoxicity against GBM tumor cell lines than CAR-T cells [11]. In this study, however, we performed a systematic comparison of the anti-tumor responses of B7H3/CD28z CAR-NK and T cells both in vitro and in vivo. We generated donor-matched B7H3/CD28z CAR-NK and CAR-T cells and compared their cytolytic activity and cytokine secretion against B7H3-positive adult GBM tumor cell lines U87 MG and A172. We observed high expression of B7H3/CD28z CAR in both NK and T cell populations in the matched donors (fig. S1A). CAR19-engineered T and NK cells and untransduced (Un)-T and untransduced (Un)-NK cells were used as controls. A 4-hour in vitro cytotoxicity assay showed that B7H3/CD28z CAR-NK cells exhibited significantly higher cell-mediated killing of U87 MG (fig. S1B) and A172 cells (fig. S1C) compared to donor-matched B7H3/CD28z CAR-T cells at all tested effector:target (E:T) ratios (p < 0.05; Fig. 1A), indicating that CAR-NK cells are more efficient at killing target cells in vitro. We also used CD19-expressing LCL lines (fig. S1E) and Raji cells (fig. S1E) as positive controls for the CAR-19-transduced NK and T cell products, and observed a similar pattern. As expected, untransduced NK cells (Un-NK) also exhibited potent cytolytic activity, consistent with our previous work. (fig. S1, B to D). We next investigated the cytolytic activity explicitly associated with CAR expression in both NK and T cells. This CAR-specific activity was calculated as the difference between the CAR-transduced T or NK cell cytolytic activity versus the control Un-transduced T or NK cell cytolytic activity. Interestingly, when we compared CAR-specific activity between NK and T cells against GBM tumor lines, the difference was tumor-dependent (Fig. 1, B and C). For example, CAR-specific T cell activity was significantly higher than CAR-specific NK cell activity at 10:1 and 5:1 E:T ratio (p < 0.05, Fig. 1B) against the U87 MG tumor line. However, in the A172 tumor cell model, when the CAR-specific activity was calculated, there was no significant difference between CAR-NK and CAR-T cells (p < 0.05, Fig. 1C). We next performed an alloreactivity test to evaluate on-target, off-tumor effects of both B7H3/CD28z CAR-transduced NK and T cells. We generated PHA blast from matched donors to assess their alloreactivity. We performed a 4-hour cytotoxicity assay to measure CAR-NK- and CAR-T-mediated killing vs. non-specific killing of donor-matched PHA blast cells. We found that CAR expression in NK or T cells did not significantly alter the alloreactive response to matched non-malignant PHA blasts relative to CAR-T cells or the untransduced (Un) controls (E:T 10:1, p = 0.7461) (Fig. S1F).

Next, we looked at IFN-γ and TNF-α secretion by NK and T cells in response to antigen stimulation using intracellular staining (ICS) (Fig. 1, C and D). B7H3/CD28z CAR-transduced NK and T cells showed an increased INF-γ- and TNF-α-positive population after stimulation with B7H3-positive U87 MG cells compared to unstimulated controls. However, B7H3/CD28z CAR-NK cells showed enhanced cytokine secretion with significant elevations in the IFN-γ level (p = 0.0359) as well as IFN-γ/TNF-α-expressing populations (p < 0.0001; Fig. 1D). In contrast, B7H3/CD28z CAR-T cells showed a significant increase only in the TNF-α-expressing population (p < 0.0001; Fig. 1D). Our study also confirms that the B7H3/CD28z CAR is highly functional in NK cells upon antigen-specific stimulation. Thus, in short-term (4 hour) cytotoxicity assay and activation studies, our results demonstrate that B7H3/CD28z CAR-NK cells exhibit potent tumor cell lysis and superior IFN-γ expression compared to B7H3/CD28z CAR-T cells. Collectively, these results indicate that either B7H3CD28z CAR-NK cells show accelerated IFN-γ secretion and target cell lysis or elicit an earlier onset of cytotoxic function (i.e., within 4 hours) compared to B7H3/CD28z CAR-T cells.

### B7H3/CD28z CAR-T cells retain potency in vivo, while NK cells fail against B7H3-expressing brain tumors

Next, we compared the antitumor efficacy of B7H3/CD28z CAR-NK and T cells in vivo, in a U87 MG murine xenograft model. We used CAR19, Un-T, and Un-NK cells as controls. 5 × 10^4 U87 MG cells were implanted intracranially. We confirmed tumor engraftment by bioluminescence imaging (BLI) five days post-tumor injection and administered 1 × 10^6 of the B7H3/CD28z CAR-NK and T cell products or controls at the same coordinates (Fig. 1E). We injected another 1 × 10^6 NK and T cell products on day 19. The untreated group (PBS) and the groups treated with Un-T cell, CAR19 T cell, and CAR19 NK cell products showed rapid tumor outgrowth after week 3, with a median survival of 48.5 days for the B7H3/CD28z CAR-NK treatment group versus 39 days for PBS treated mice, 43 days for the CAR19-NK treatment group, and 39.5 days for the Un-NK treatment group. In contrast, the B7H3/CD28z CAR-T cell treatment group showed no tumor growth up to week 15, the duration of the observations. The median survival of B7H3/CD28z CAR-T-cell-treated mice was significantly higher than that of respective controls (Fig. 1F; p < 0.05). Median survival was 39, 41.5, and 35 days in the PBS, UT, and CAR19-T treatment groups, respectively, while median survival was not reached for the B7H3/CD28z CAR-T treatment group by the end of the 137-day follow-up period. Hence, despite B7H3/CD28z CAR-NK cells showing accelerated cytotoxicity compared with B7H3/CD28z CAR-T cells in a short-term killing assay, they failed to restrict tumor growth or improve overall survival *in vivo* (Fig. 1, E and F). Therefore, we next performed ddPCR on brain and spleen tissues to verify whether the CAR-expressing NK versus T cells persisted *in vivo*. The CD45AP primer was used to detect both T and NK cells, and a primer specific to the CD28 transmembrane domain was used to identify the expression of both CARs (B7H3 and CAR19). We detected high levels of CAR and CD45 expression in brain samples derived from T- and NK cell-treated mice that were sacrificed at 40–60 days (due to terminal disease) (fig. S1G). But in mouse groups sacrificed at the end of the study, we observed low levels of CD45 and CAR expression (B7H3/CD28z CAR-T cell group and one mouse from the B7H3/CD28z CAR-NK group), which could be attributed to effector cell loss due to limited proliferation in the absence of tumor antigen. Compared to the spleen, we observed higher levels of CAR and CD45 signals in the brains of mice, indicating greater localization in the tumor-bearing B7H3-positive brain area (fig. S2B). Based on these observations, we conclude that the B7H3/CD28z CAR-NK-treated mouse group showed sustained CAR and CD45 expression for a significant period (40–60 days post-tumor engraftment), suggesting that NK cells persisted while tumor growth remained unaffected.

We also confirmed NK cell infiltration into the tumors using immunofluorescence (fig. S2). CD45 immunofluorescence staining was performed on brain tissue collected at the study endpoint to detect human immune cells. Images were acquired using an Evident Scientific VS200 slide scanner, and tumor regions were identified by mCherry fluorescence (fig. S2E–F). CD45^+^ cells co-localized within those tumor regions, indicating that NK cells infiltrated the tumors (fig. S2E–F). After establishing NK cell infiltration and persistence at the tumor site, we next investigated additional mechanisms that may contribute to the B7H3/CD28z CAR-NK cell dysfunction in vivo, including TGF-β–mediated immunosuppression, B7H3 antigen modulation (including upregulation on effector cells and trogocytosis), and NK cell exhaustion.

### TGF-β-mediated Immunosuppression

We previously showed that TGF-β secreted by GBM rendered the CAR-NK cells nonfunctional, leading to downregulation of activation receptors and significantly reducing NK cell cytolytic activity in U87 MG cell line [11]. To overcome this, we transduced NK cells to co-express a TGF-β dominant-negative receptor (DNR)[14] with the CAR (Fig. 2A). We evaluated our DNR.B7H3 CAR-NK product in vitro against a patient-derived (PDX) GBM cell line, GSC-272. We used GSC-272 in addition to U87 MG in order to evaluate CAR-NK activity in a patient-derived glioblastoma stem-like model that retains stem-like properties and more closely reflects clinically relevant GBM biology (Fig. 2B and fig. S3)[15, 16]. Importantly, both U87 MG and GSC-272 tumor lines are known to secrete TGF-β [11, 16]. Co-expression of TGF-β-DNR and CAR on NK cells protected the cytolytic function in the presence of TGF-β [11]. We found no significant difference in the cytolytic potency of DNR.B7H3 CAR-NK-cells in the presence or absence of TGF-β at all E:T ratios (p > 0.05), while NK cells transduced with B7H3 CAR without DNR showed a significant decrease in their cytolytic activity upon exposure to TGF-β (E:T ratio 10:1; p = 0.02857) (Fig. 2B). Next, we evaluated our DNR.CAR-NK’s antitumor response *in vivo* using the U87 MG murine model. We used B7H3/CD28z CAR, DNR, and Un-NK cells as controls. 5 × 10^4 U87 MG cells were implanted intracranially, and two doses of 2 × 10^6 NK cell products were administered at the same coordinates, on day 5 and on day 10. We adjusted our second injection schedule according to the tumor relapse timing observed in our previous in vivo study, with the goal of administering the second dose before relapse occurs. All mice, including untreated (PBS) and NK cell-treated groups, showed rapid tumor outgrowth (Fig. 2C). The median survival of DNR.B7H3 CAR-NK cell product-treated mice was 32 days which was comparable to that of B7H3.CD28z-CAR NK (39 days), DNR-NK (36.5 days), Un-NK (39 days), and PBS (39 days) treatment groups (Fig. 2E; p > 0.05). These results indicate that the DNR-CAR-NK cell product still failed to confer greater murine survival than other conditions. Although we confirmed *in vitro* that DNR can salvage CAR-NK cells’ cytolytic activity in the presence of exogenous TGF-β, we observed no effect of DNR when added to the B7H3/CD28z CAR-NK cell product *in vivo*. However, since we used an NSG xenograft model that does not accurately recapitulate TGF-β-mediated immunosuppression in GBM tumors, we cannot definitively conclude that the DNR was ineffective in vivo. Therefore, we chose to explore other mechanisms that may underlie NK cell failure.

### B7H3 antigen expression is high in activated NK cells

B7-H3 belongs to the immune co-inhibitory B7 superfamily, is associated with poor prognosis, and is known to inhibit NK and T cells.[17] The human B7-H3 protein is expressed at very low levels in NK and T cells but can be induced upon activation [11, 12], and may lead to activated CAR-NK cells engaging one another (fratricide) rather than with the target tumor cells. Therefore, we next investigated B7H3 antigen expression in NK and T cells during *ex vivo* expansion. We observed that both NK and T cells upregulated B7H3 expression (Fig. 3A). On day 15, when we typically harvest the cells for in vivo evaluation, B7H3 antigen expression was similar between B7H3/CD28z CAR-NK cells (21%±3.97%) and Un-NK cells (23.4%±10.53%) (p = 0.9727; fig. S4A). Meanwhile, B7H3 antigen expression on B7H3/CD28z CAR-T cells (25.15%±5.42%) was significantly higher than Un-T cells (6.64%±2.55%) (p = 0.0078) but comparable to B7H3/CD28z CAR-NK cells (p = 0.8772, fig. S4A). As shown above, B7H3/CD28z CAR-T cells have a robust antitumor response in vivo. This suggests that B7H3 antigen upregulation on NK cells by itself is unlikely to be the primary reason for their failure in vivo. To further elucidate whether B7H3 antigen upregulation contributes to the limited in vivo activity of NK cells, we disrupted endogenous B7H3 expression in NK cells using CRISPR-Cas9. Knockout (KO) of B7H3 antigen on the NK cells’ surface was confirmed by flow cytometry and KO Inference of CRISPR Edits (ICE) analysis (Fig. 3B and fig. S4B). While we observed a decrease in B7-H3 expression in Un-NK cells, KO-CAR-NK cells still showed B7-H3 on the surface, which was comparable to that in control CAR-NK cells (Fig. 3C and fig. S4C). Therefore, we sought an alternative explanation for the detection of B7H3 antigen on CAR-NK cells even after B7-H3 knockout: trogocytosis.

### NK cell fratricide is minimal despite B7H3 antigen trogocytosis

Trogocytosis is a phenomenon in which an antigen is transferred from the tumor surface to lymphocytes, especially NK cells, during cell-cell contact through the transfer of the broken target cell membrane. Trogocytosis by NK cells is currently under extensive investigation [18, 19]. If trogocytosis occurs in B7H3 CAR-NK cells, it may explain why they retain the B7-H3 antigen even after KO. Specifically, trogocytosis of the targeted antigen from tumors to CAR-NK cells can lead to fratricide and result in CAR-NK cell failure. To verify trogocytosis of the B7-H3 antigen onto NK cells, we cocultured CAR-NK cells and B7H3-KO CAR-NK cells with B7-H3-expressing tumor cell lines, and then stained them for B7-H3 antigen. We observed a higher expression level of B7-H3 antigen on the surface of both B7H3/CD28z CAR-NK and B7H3-KO-CAR-NK cells (fig. S4D), indicating that these NK cells undergo trogocytosis. We then investigated whether trogocytosis-driven fratricide occurs with B7H3/CD28z CAR-NK cells. We cocultured B7H3/CD28z CAR-NK and Un-NK cells with and without U87 MG cells. In one set of experiments, LAT-A inhibitor was included as a negative control because it inhibits synapse formation. We stained the NK cells with Cell Trace Violet (CTV) and used eTox Green (Agilent Technologies) to identify dead cells throughout the experiment. During analysis, we gated on CTV and green double-positive cells to quantify the dead NK cells in culture. We observed very minor NK cell death when cultured with targets. The maximum NK cell death observed after 20 hours when CAR-NK cells where cocultured with U87 MG cells was 4.8% ± 2.124% versus 2.226% ± 0.656% when Un-NK cells were co-cultured with U87 MG cells. There was a significant decrease in the percentage of NK cell death between groups (Fig. 3D): NK+U87 vs NK+U87 + LAT-A; CAR-NK+U87 vs CAR-NK+U87 + LAT-A, which could be attributed to a decrease in the eToxGreen signal at late time points, when dead cells begin to break apart completely. Overall, these studies show no significant increase in CAR-NK or Un-NK cell death when cocultured with the target, indicating that, regardless of B7H3 antigen trogocytosis, the fratricide is not significant.

Next, we hypothesized that the lack of fratricide, despite observing B7H3 antigen trogocytosis, may be attributed to the higher antigen density on tumor cells relative to NK cells, even after antigen acquisition by NK cells through trogocytosis. We also verified the overall change in B7H3 MFI (Median Fluorescence Intensity) due to B7H3 antigen transfer from the target to the effector (NK or T cells) during coculture (Fig. 3E and fig. S4F). We used B7H3-CAR-T cells as a control. Our results show that the change in B7H3 MFI in CAR-NK cells after coculture with B7H3^+^-target cells, compared with CAR-NK alone, was not significant (p > 0.05). When we examined changes in B7H3 MFI in tumor cells under coculture vs. tumor-only conditions, we found no significant difference in B7H3 MFI between these conditions (fig. S4G). Thus, we conclude that the increase in B7H3 antigen expression on B7H3/CD28z CAR-NK cells, due to trogocytosis, was insignificant to trigger fratricide, as the antigen density on the GBM tumor is substantially higher (Fig. 3E).

### Repetitive engagement with tumor antigen drives NK cell exhaustion

After confirming that trogocytosis-driven fratricide does not explain the failure of B7H3/CD28z CAR-NK cells to successfully treat U87 GBM tumors *in vivo*, we evaluated whether functional exhaustion of NK cells leads to their dysfunction in this model. We performed serial rechallenges with the same tumor cells, to try and recapitulate the in vivo conditions, and tested the potency of CAR-NK cells after each challenge. In this assay, B7H3/CD28z CAR-NK cells lost their killing activity after the second stimulation, whereas B7H3/CD28z CAR-T cells retained their killing ability even after the fourth round of stimulation (the maximum number of rechallenges performed). B7H3/CD28z CAR-NK cells’ cytolytic activity was low and comparable to that of the Un-NK cells (p > 0.05) and significantly less than that of B7H3/CD28z CAR-T cells after the 1st and 2nd tumor stimulations (p = 0.002165) (Fig. 4A). When we examined their proliferation after each tumor challenge, B7H3/CD28z CAR-NK cells were comparable to CAR-T cells after the 1st tumor challenge (p = 0.55) but their relative proliferation decreased significantly after successive challenges (p = 0.008) (Fig. 4B). We also investigated their cytokine secretion after each tumor challenge. We collected supernatant 24 hours after each stimulation and performed ELISA to quantify IFN-γ and TNF-α. IFN-γ secretion levels for B7H3/CD28z CAR-NK cells (514.24 ± 20.75 pg/ml) and B7H3/CD28z CAR-T cells (524.61 ± 0.0006 pg/ml), which were above the saturation signal after the 1st tumor challenge. However, after the 2nd challenge, IFN-γ secretion level dropped significantly in B7H3/CD28z CAR-NK cells compared to B7H3/CD28z CAR-T cells (p < 0.028) (Fig. 4C). B7H3/CD28z CAR-T cells consistently secreted high levels of IFN-γ, even after the 4th tumor challenge. TNF-α levels secreted by B7H3/CD28z CAR-NK cells (49.12 ± 41.27 pg/ml) were also dramatically less than that of B7H3/CD28z CAR-T cells (saturation at 1049.21 ± 0.01 pg/ml) after the 1st and 2nd tumor challenges (p < 0.029) (Fig. 4D). Thus, these studies show that the proliferation and cytokine secretion of NK cells are significantly reduced compared to T cells, consistent with functional exhaustion.

Next, we tested whether functional exhaustion was impacted by CAR expression or the inherent activity of NK cells. Therefore, we evaluated CAR19-NK cells in the tumor-rechallenge model with the CD19 + Raji tumor cell line and observed a similar exhaustion pattern, with decreased serial tumor killing following repeated antigen stimulation, proliferation, and cytokine secretion for both CAR19-NK cells and untransduced NK cells. Moreover, CAR19-NK cells also showed functional exhaustion, as evidenced by reduced cytotoxic activity following repeated tumor challenge against CD19 + Raji cells (fig. S5A) with decreased secretion of IFN-γ and TNF-α (fig. S5B) compared to CAR19-T cells. These studies indicate that the loss of effector function, i.e., cytolytic activity, is due to the NK cell component rather than the CAR construct per se.

We also investigated whether the expression of any of the functional markers is altered, which could explain why NK cells stop proliferating and cease killing following repeated tumor challenges (n = 3). We used OMIQ software to visualize flow cytometry data in high-dimensional space by Uniform Manifold Approximation and Projection (UMAP) and t-distributed Stochastic Neighbor Embedding (tSNE) (Fig. 4.E). We observed that all unstimulated cells (T = 0) cluster together and are very distinct from stimulated/exhausted NK cells (T = End point). We observed a number of clusters, including 2, 4, 7, 10, 12, 14, 19, and 23, which were exclusive to unstimulated NK cells, and 20 and 21, which were exclusive to stimulated NK cells (Fig. 4H and Table 1). Figure 4E summarizes the change in NK receptor expression, both in Median Fluorescence Intensity (color scale) and Percentage/Fraction (circle size). The percentage change in NK receptor expression between stimulated and unstimulated NK cells were visualized using a volcano plot, with log_2_ fold change plotted on the x-axis and − log_1__0_ adjusted p-value on the y-axis (Fig. 4G). We observed a significant decrease in activating receptors (NKp46 and NKG2D) and LAG3, and a significant increase in TIGIT and NK cell inhibitory receptors (NKG2C and KIR2DL2/3) in exhausted NK cells (Fig. 4G). We also observed a decrease in DNAM and FAS MFI following tumor stimulation. The downregulation of DNAM in activating receptors has crucial implications in the U87 GBM model we are evaluating since U87 MG tumor cells express PVR, a DNAM receptor ligand [10]. Collectively, these results reveal that NK cells have a novel tumor-escape mechanism that contributes to resistance to NK cell-mediated immunotherapy in the aggressive U87 GBM model.

### IL-15 can rescue NK cells from functional exhaustion

To address CAR-NK exhaustion and tumor resistance, we reevaluated the NK cell in tumor rechallenge model (Fig. 5A). The cytokine IL-15 has previously been shown to promote NK cell proliferation and rescue them from functional exhaustion [20–22]. When we added IL-15 to the NK cell culture, both B7H3/CD28z CAR-NK and Un-NK showed enhanced proliferation and effective tumor lysis (Fig. 5B and fig. S5C). IFN-γ cytokine secretion by NK cells after each tumor stimulation also exceeded the saturation signal (524.61 pg/ml). We further confirmed this phenomenon with the CAR-19-NK cell product, where IL-15 efficiently restored antitumor activity, proliferation, and cytokine secretion (IFN-γ saturation) *in vitro*. IL-15 also improved the CAR-19-NK cytolytic function and proliferation across multiple rounds of tumor (Raji cells) stimulation (fig. S5D-E). With these successful results, we next generated IL-15-expressing CAR-NK cells to overcome exhaustion driven by repetitive antigen engagement in NK cells (Fig. 5C). We performed repeated tumor-killing assays with the IL-15-expressing NK product against the U87 MG tumor model *in vitro*. As shown in Fig. 5D, IL-15 expression enhanced CAR-NK cells’ repetitive tumor killing of U87 MG tumor cells.

We next validated the IL-15-engineered NK cell product in vivo. NK cells engineered to secrete IL-15 elicited a dramatic decrease in tumor progression at six days post-infusion (Fig. 5E–F). Unfortunately, despite the successful anti-tumor response demonstrated with IL-15 engineered CAR-NK cells, the survival analysis revealed no significant benefit between the treatment groups (Fig. 5E). Mice injected with IL-15-engineered NK cells still succumbed at a rate similar to tumor-bearing mice, and this death could be attributed to IL-15 toxicity, rather than to GBM tumors, as indicated by the observed weight loss in these mice (Fig. 5G). We also examined whether adding an NK cell-specific costimulatory domain to the CAR construct could confer a functional advantage by reducing immune dysfunction triggered by repeated stimulation. Based on published reports [23] and our experience, we designed a B7H3 CAR with a 2B4 costimulatory domain (fig. S6A). We then performed a repetitive tumor-stimulation experiment with these B7H3.2B4 CAR-NK cells, but these NK cells also failed after the 2nd tumor cell stimulation (fig. S6B). Thus, unlike findings in other tumor models—such as atypical teratoid/rhabdoid tumors [24]—we did not observe enhanced serial killing capacity with these B7H3.2B4 CAR NK cells in our U87 MG GBM model (fig. S6B).

### NK cells and CAR-NK cells administered in a low-tumor-burden setting elicit potent anti-tumor activity

Although CAR-NK cells demonstrated potent anti-tumor cell activity in vitro, these effects are not reproducible in our established in vivo brain tumor model using the aggressive U87 cell line. Our findings suggest that this limited in vivo activity of NK cells is multifactorial, including immunosuppression by TGF-β and features consistent with cellular exhaustion, which collectively diminish NK cell cytotoxic function *in vivo*.

We further demonstrate that the expression of a dominant-negative TGF-β receptor (DNR) attenuates TGF-β signaling and restores NK cell cytolytic activity in the presence of TGF-β. Next, we hypothesized that frequent NK cell administration in a low-tumor-burden disease setting may help overcome this limitation. Thus, we redesigned our in vivo model (Fig. 6A and E) to exploit NK cells’ rapid tumor-killing kinetics while compensating for their limited capacity for serial killing by implementing an early, intensified NK cell dosing schedule. Remarkably, early administration of NK cells conferred a survival advantage to all animals receiving NK cell products, including untransduced (Un) NK, DNR-NK, and DNR.B7H3 CAR-transduced NK cells (Fig. 6B–C). Further, the in vivo potency of Un-NK cells was comparable to that of the genetically engineered NK cell products. We then verified whether the NK cell products infiltrate the tumor and persist in vivo. The DNR and B7H3 CAR transgenes were detected by ddPCR in brain tumor samples collected at the study endpoint, indicating the infiltration and persistence of NK cell products in the tumors (Fig. 6D–E). Next, we examined the KIR and HLA types of both the target and the effectors to understand the unexpectedly elevated activity of Un-NK cells. HLA typing results indicated that the donor was mismatched with the tumor lines (Table 1) and the KIR profiling shows multiple matched activating receptors on donor NK cells (Table 2). This expression pattern suggests a potentially enhanced activation profile, which may contribute to the observed functional responses of NK cells irrespective of gene engineering in our experimental model.

## DISCUSSION

In this work, we sought to address the challenges of developing an allogeneic “off-the-shelf” NK-cell-based immunotherapy for GBM using the highly aggressive U87 model. We assessed a strategy of redirecting NK-cell specificity to the tumor-associated antigen B7H3 using a chimeric antigen receptor (B7H3-CAR) to enhance NK-cell potency against GBM tumors. To evaluate the potency of the CAR-NK cells, we conducted a side-by-side comparison with CAR-T cell products generated from the same donors. We found that B7H3.28z-CAR-NK cells induced robust killing of GBM cells in vitro, similar to B7H3/28z-CAR-T-cells. However, in the in vivo U87 MG model of aggressive adult GBM, CAR-NK cells failed to prevent tumor progression and improve survival, where CAR-T cells retained full potency. Hence, we interrogated multiple mechanisms that might cause B7H3-CAR-NK cell failure in this setting and sought multiple solutions in a stepwise manner.

First, we investigated immunosuppression in the TME of GBM, which dampens CAR-NK cell activity. We previously showed that TGF-β secreted by the GBM cell line U87 MG created an immunosuppressive microenvironment that rendered CAR-NK cells dysfunctional.[11] Therefore, we used a dominant negative TGF-βRII (DNR) to block its activity [25]. While NK cells engineered to express the DNR elicited positive and unhindered cytolytic activity in the presence of TGF-β *in vitro*, B7H3 CAR NK cells expressing the DNR did not improve survival compared with other NK cell products *in vivo*. Nevertheless, we cannot definitively conclude that incorporating DNR into NK cells was ineffective, given that NSG mice are not an optimal in vivo model for evaluating TGF-β-mediated immunosuppression. Moreover, the lack of suitable alternative in vivo models (e.g inducible TGF-β knockout systems) in humanized GBM models makes this a challenging question to fully answer.

Next, we observed that NK cells expressed a higher level of B7H3 antigen upon activation than at the time of injection. Believing that NK cells upregulate B7H3 expression and commit fratricide, we knocked out (KO) B7H3 antigen in NK cells. Even after B7H3 KO, the B7H3 antigen level on CAR-NK cells remained high. We sought an alternative explanation for the increase in B7H3 antigen level: Trogocytosis, a process in which NK cells might acquire this antigen from target tumors upon cell-cell contact. We confirmed that the B7H3 CAR-NK cells underwent trogocytosis of the B7H3 antigen following stimulation with B7H3^+^ U87 cells. However, it did not lead to significant fratricide in our CAR-NK cells when cocultured with the GBM U87 MG cell line. Upon further investigation, we observed that B7H3 antigen density on CAR-NK cells is significantly lower than on the U87 MG target tumor cells. Notably, the overall B7H3 MFI on effectors remains markedly lower than on U87 MG target tumor cells, supporting our hypothesis that the MFI increase on effectors is insufficient to trigger fratricidal activity (Fig. 3E).

We then explored whether cellular exhaustion contributes to NK cell suboptimal performance *in-vivo*. We used a tumor-repetitive stimulation model, in which repeated engagement of the target antigen can cause functional exhaustion. We observed that CAR-NK cells undergo functional exhaustion soon after the 1st exposure to tumors with a progressive decline in their tumor-killing efficacy, as evidenced by downregulation of activating receptors, reduced target cell lysis, diminished cytokine secretion, and impaired proliferative capacity. CAR-T cells, on the other hand, maintained their full potency even after the 4th challenge. These observations could explain the tumor resistance *in vivo* observed with our B7H3/CD28z CAR-NK products and their underperformance compared to CAR-T cells. Thus, this study represents the first to characterize the exhaustion phenotype in CAR-NK cells in the aggressive preclinical GBM U87 model, which enables immune evasion and tumor escape. IL-15 has been known to rescue NK cells from metabolic exhaustion while promoting their proliferation [20–22]. Therefore, we tested whether IL-15 inclusion could overcome NK cell functional exhaustion in the GBM U87 MG model. With IL-15 supplementation, CAR-NK cells retained cytolytic activity after repeated tumor rechallenges and demonstrated tumor-specific killing effects comparable to those of CAR-T cells. Moreover, IL-15 rescued NK cells’ effector functions, leading to inhibition of tumor growth *in vivo*. Unfortunately, when delivered directly into the CNS, IL-15 itself induced toxicity, which negatively affected overall survival.

Although CAR-NK cells showed superior, rapid tumor cell killing *in vitro* compared to CAR-T cells, translating these results to an *in vivo* setting in an aggressive GBM model has encountered many obstacles, and our solutions to these issues seem to introduce new problems. Therefore, we decided to leverage NK cells’ rapid action and overcome functional exhaustion by administering the therapeutic B7H3/CD28z CAR-NK product multiple times. In fact, with early and intensified NK cell dosing, we achieved dramatic improvements in survival outcomes compared with untreated controls. These results reveal for the first time that frequent delivery of NK cells at early time points sustains NK cell activity even through repeated tumor antigen exposures that cause exhaustion and functional impairment. However, surprisingly, the untransduced (Un)-NK cell group also showed enhanced survival and even outperformed the gene-engineered CAR-NK product. Further investigation showed that the enhanced tumor killing by Un-NK cells correlated with the MHC mismatch and HLA match of the NK cell product to the tumor cell line. Our results agree with previously published studies in the hematopoietic stem cell transplant (HSCT) setting, where MHC-mismatched HSCT (specifically HLA-haploidentical HSCT) with KIR-ligand mismatch has been a powerful strategy that leverages NK cell-mediated graft-versus-leukemia to improve leukemia-free survival, reduce relapse, and lower non-relapse mortality [26, 27]. Immune rejection is a major barrier to NK cell therapy in allogeneic settings, reducing the persistence of adoptively transferred cells and potentially limiting clinical efficacy.[28–30] Approaches to address this include engineering NK cells to evade host immunity by expressing HLA-E, HLA-G, or CD47, or by deleting MHC class I and II molecules [31–33]. However, our model, with repeat NK cell dosing in a CNS tumor setting, will support sustained NK cell presence at the tumor site, potentially overcoming this limitation. Collectively, our data suggest that NK-cell immunotherapy may be most effective for GBM when administered early, before tumor burden escalates. This suggests that NK cell immunotherapy should be administered early post standard of care treatment, at a time of minimal residual disease.

Few orthotopic U87 MG models have been published, aiming to harness the therapeutic potential of NK cells. *Morimoto et al*. observed growth inhibition by retro-orbital venous injection of NK cell products, but provided no evidence of tumor clearance [34]. *Tran et al*. studied the orthotopic U87 MG model, but their reported survival data are from a subcutaneous tumor engraftment model and not a CNS model [35]. Similarly, *Shida et al*. reported only prolonged survival in the subcutaneous U87MG-derived tumors. In other studies, therapeutic gene-engineered (e.g., CAR or cytokine-like IL-21) NK cell products have shown success in GBM patient-derived xenografts [36, 37]. For example, *Choe et al*. recently demonstrated extended survival in orthotopic xenografts using the B7H3 CAR-NK product in atypical teratoid/rhabdoid tumors [24]. This may suggest that the limited in vivo response of the CAR-NK cells in the adult GBM U87 MG tumor model we used is attributable to the tumor’s aggressive nature; therefore, we assert that in this model, the utility of the NK cell platform may be most effective in a low-tumor-burden setting. We also acknowledge that immortalized cell lines, such as U87 MG, grow rapidly in mice and may not accurately predict treatment response, and that employing patient-derived xenografts may provide additional potentially clinically relevant insights and will be the focus of our future studies.

In conclusion, our study compares T and NK cell therapeutic derivatives side-by-side in identical CAR-engineered settings to more accurately reflect the functional differences between the two effectors. While CAR-NK cells showed rapid action and greater potency against tumor cells in vitro, CAR-T cells showed superior antitumor responses in vivo owing to their resistance to senescence and their ability to sustain function during repeated antigen engagement. We demonstrated that CAR-NK cell dysfunction in the aggressive U87 GBM model was multifactorial, including immune suppression and NK cell exhaustion following repeated tumor engagement. We attempted to negate TGF-β-mediated immunosuppression and NK-cell dysfunction with TGF-β DNR and overcome functional exhaustion of CAR-NK cells by including IL-15 in the therapeutic design, but both approaches failed to improve survival outcomes. Finally, we modified the NK cell administration protocol to mitigate these constraints by delivering NK cells early and frequently, a strategy that proved successful. Our findings have direct implications for allogeneic NK cell therapy in the treatment of aggressive solid tumors, indicating a survival advantage of early NK cell administration before tumor burden escalates or in the context of minimal residual disease. Additionally, our results also reveal that NK cells may achieve sufficient therapeutic efficacy without gene engineering in select tumor contexts if donor KIR-HLA matching is considered.

## MATERIALS AND METHODS

### Cell lines and mouse models

U87 MG cells were purchased from American Type Culture Collection (ATCC, Manassas, VA) and were cultured in Gibco DMEM:F12 media (1:1) (Thermo Fischer Scientific, Waltham, MA) with 10% Hyclone FBS (Cytiva, Logan, UT), and 1% Gibco Glutamax (Thermo Fischer Scientific, Waltham, MA), and were grown in humidified incubators at 37°C with 5% CO2 air. GSC-272, established from GBM tumor samples, was kindly provided by Dr. Frederick Lang, MD Anderson Center, Houston, TX. GBM-110 FHTC primary pediatric cancer cell lines were purchased from Fred Hutchinson Cancer Research Center. GSC272 and GBM110 tumor cell lines were grown in defined serum-free neural stem cell (NSC) medium: DMEM/F12 (Corning Inc., NY) supplemented with B-27^™^ Supplement (50×, Gibco, Thermo Fisher Scientific, Waltham, MA), EGF, and FGF (Shenandoah Human Growth Factors, Warwick, PA) on laminin-coated plates (Thermo Fisher Scientific, Waltham, MA). Raji cells were purchased from ATCC and cultured in Gibco^™^ RPMI 1640 Medium (Thermo Fisher Scientific) supplemented with 10% Hyclone FBS (Cytiva, Logan, UT).

Male and female NSG mice (4–6 weeks old) were purchased from the Jackson Laboratory. All mice were housed in specialized, high-barrier facilities (SPF) in the Animal Core Facility at Children’s Hospital. Mice were maintained on a 12-hour light/dark cycle with controlled temperature and humidity and provided ad libitum access to autoclaved (or irradiated) standard rodent chow and sterilized drinking water. All mouse studies were performed in accordance with the Children’s National Hospital Animal Husbandry guidelines, and the experimental protocols were approved by the Institutional Animal Care and Use Committee (IACUC).

### Expansion of NK cells and viral transductions

NK cells were expanded from cord blood MNCs (Stem Cell Technologies, Cambridge, MA) or peripheral blood buffy coat from the Gulf Coast Regional Blood Center, Houston, TX, using an NK cell isolation kit (Stem Cell Technologies). Isolated NK cells were stimulated on day 0 with irradiated (100 Gy) K562-based feeder cells, express**ing** 4–1BB ligand and membrane bound IL-15 (Kindly donated by Dario Campana), at a feeder cell**s**:NK ratio of 2:1, and recombinant human IL-2 (Proleukin, 200 IU/ml; Chiron, Emeryville, CA) and IL-21 (R&D Systems) in complete CellGenix GMP SCGM Stem Cell Growth Medium (CellGenix GmbH, Freiburg, Germany) as described earlier [38], and were transduced with B7H3/CD28z CAR (SFG backbone) retrovirus or CAR19/CD28z (control), kindly provided by Dr Barbara Savaldo from UNC, by spinoculation at 2000 g for 2 hrs [12, 39]. DNR retroviral supernatant was generated using PG13 producer cells, as previously described.[25] CAR-T cells were generated by spinoculation of activated MNCs according to the established protocol.[12]

### Short-term cytotoxicity assay (4-hour)

The Celigo Imaging Cytometer-based cytolytic assay was performed for 4 hours to determine the cytolytic activity of transduced NK cells [40, 41]. Briefly, B7H3^+^ targets were labeled with Calcein AM dye (Thermo Fisher Scientific), and target cells were cocultured with transduced and untransduced control NK cells (UT-NK) in a flat-bottom microplate at E:T ratios of 10:1, 5:1, and 1:1. Cells were imaged after 4 hrs using the Celigo Imaging Cytometer system, and cell lysis was quantified based on the decrease in Calcein-positive cells, as dead cells lose the calcein expression.

### Tumor rechallenges, luciferase cytotoxicity assay, and xCELLigence killing assays

mCherry-expressing tumor cells were seeded onto a 96-well white solid-body flat-bottom plate (Thermo Scientific^™^) and incubated for 1 hour to allow adhesion. Effector cells were then added at an Effector-to-Target (E:T) ratio of 2:1, while control wells contained only tumor cells in media and 1% Triton X-100. After 2–4 days of exposure, NK cells from the killing assay plates were transferred to newly seeded tumor cells for rechallenge. Tumor cell growth was measured by adding D-luciferin (GoldBio, St. Louis, MO) at 75 μg/ml and quantifying luciferase activity [42, 43]. The plates were dark-adapted for 5–10 minutes in a plate shaker and read using the Tecan plate reader (US Inc., Morrisville, NC). Effector cells were rechallenged until they were exhausted or for up to 4 tumor stimulations.

For xCELLigence killing assays, mCherry-expressing tumor cells were seeded onto a 96-well flat-bottom TC-treated plate (Corning Inc.) and rechallenges were carried out as above under different experimental conditions. mCherry and bright-field images were taken at 180-minute intervals over several days (either when effectors were exhausted or after a maximum of 4 tumor stimulations) using the xCELLigence Real-time Analysis System (RTCA) (Agilent Technologies, Santa Clara, CA). Tumor cell growth was measured by quantifying the mCherry signal remaining in the plate and expressed as a percentage of cytolysis.

### ELISA

INF-γ and TNF-α cytokine levels were measured in supernatants collected 24 hours after coculture was established, using ELISA MAX^™^ Deluxe sets for human IFN-γ and TNF-α (BioLegend, San Diego, CA) following the manufacturer’s instructions. Samples were read using a Tecan plate reader (US Inc., Morrisville, NC, USA). We reported saturated values for some conditions due to limited samples that prevented dilution and repeat testing.

### Flow cytometry

Flow cytometry was used to assess NK cell phenotype with antibodies listed in the Supplementary Table. Expression of the B7H3 CARs was detected using a two-step staining protocol. CAR NK cells were stained with recombinant human B7H3 Fc chimera protein purchased from R&D Systems and then stained with secondary F(ab’)_2_ fragment of goat anti- human IgG (H + L) (Jackson ImmunoResearch, West Grove, PA) to identify B7-H3 protein. The staining intensity of samples was acquired using a CytoFLEX Research Flow Cytometer (Beckman Coulter, Brea, CA) or Cytek Aurora flow cytometer (Cytek Biosciences, Fremont, CA). For each sample, a minimum of 10,000 events were acquired, and data were analyzed using FlowJo v10 (BD Biosciences, Ashland, OR) or OMIQ (Dotmatics, Boston, MA), as described in the figure legend. We followed the following gating strategy for samples in OMIQ: Cells◊Singlets◊Live◊mcherry-◊CD3-CD56 + then subsampled to 30,000 CD3-CD56 + events to standardize across all samples. Next, we performed t-distributed stochastic neighbor embedding (t-SNE) followed by FlowSOM clustering analysis and visualization (FlowSOM: Using self-organizing maps for visualization and interpretation of cytometry data - Van Gassen − 2015 - Cytometry Part A - Wiley Online Library). Finally, relative MFI of each marker and cluster is assessed using edgeR to establish an increase or decrease in relative expression. All antibodies used are summarized in Table 2. Finally, the clustering data were exported from OMIQ and uploaded and analyzed using Microsoft Copilot to help identify cluster subsets based on the NK cell markers expression.

### Xenogeneic mouse models

U87 MG mCherry FFlyluc tumor cells were suspended at 50×10^3 cells/mL in DPBS, and 4 μL of the cell suspension was surgically implanted into the brains of 5- to 8-week-old male NOD mice Cg-Prkdc scid Il2rg tm1Wjl/SzJ (NSG) using a 26sG Hamilton syringe (Part # 80008) at the coordinates x: −2.5mm (median lateral), y: −3.5mm (dorsal ventral), and z: −0.5mm (Anti-posterior) from bregma. The wound was closed by using stainless steel autoclips (Braintree Scientific, Braintree, MA). Weekly bioluminescence imaging was used to track tumor progression by IVIS Lumina 100 (PerkinElmer, Waltham, MA). Using Living Image software, the photon flux rate within standardized regions of interest (ROI) was calculated to quantify the signal in photons/second (p/s). On days 5 and 19 following tumor cell implantation, or as indicated in the figure legends, NK cells (1×10^6^ or 3×10^6^ NKs in 4 μL) were injected intracranially at the same stereotactic coordinates. Tumor growth will be monitored twice weekly using the Xenogen-IVIS Imaging System. Mice showing signs of discomfort, weight loss (more than 20%), neurological symptoms (e.g., seizures), or that were moribund were euthanized according to IACUC guidelines. Brain and other tissues were collected for immunohistochemistry, or their DNA and RNA were extracted and stored at −20°C for later ddPCR use.

### ddPCR protocol

RNA or genomic DNA was extracted from mouse tissue or cells according to the manufacturer’s instructions (Zymo Research, Irvine, CA). RNA samples were reverse transcribed using the iScript cDNA Synthesis Kit (Bio-Rad Laboratories, Hercules, CA). B7H3 CAR primers, previously published by Haderbache et al. [27], DNR primers, from our group’s previous publication [44], and pre-made primers for mouse Actin-B (positive control) and GFP (negative control), were obtained from Integrated DNA Technologies, San Diego, CA. CD45AP pre-made primers (Bio-Rad Laboratories) were used to detect lymphocytes. The ddPCR master mix was prepared with 10 μL of QX200^™^ ddPCR^™^ EvaGreen Supermix (Bio-Rad Laboratories), 100 ng of gDNA or cDNA, 150 nM each of forward and reverse primers, and nuclease-free water, in a total volume of 20 μL. The mixes were loaded into the DG8^™^ Cartridge (Bio-Rad Laboratories), and droplets were generated using the QX200^™^ Droplet Generator (Bio-Rad Laboratories). The droplet emulsions were transferred to a PCR machine and cycled according to the following thermal cycler settings: 95°C for 5 minutes, 40 cycles at 95°C for 30 seconds, 60°C for 1 min, and 4°C for 5 min, and a final step at 90°C for 10 minutes, followed by 4°C for 5 minutes. Data acquisition and analysis were performed with the QX200^™^ Droplet Reader and QuantaSoft^™^ software (Bio-Rad Laboratories).

### CRISPR gene editing

B7H3 sgRNA guides were designed using Synthego CRISPR Design Tools. The designed sgRNAs and Cas9 were obtained from Synthego, Redwood City, CA (1.5 nmol). The following sgRNAs were used to create B7H3 knockouts.

UGCCCACCAGUGCCACCACU

GUGCCCACCAGUGCCACCAC

AAAGCUGUGCACCAGCUGUU

UUUGCUGAGGGCCAGGACCA

The sgRNAs were resuspended in nuclease-free TE buffer at a concentration of 100 μM, and then combined with Cas9 in Nucleofector buffer (Lonza Electroporation Kit, Lonza, Basel, Switzerland) at a ratio of 3:1 of sgRNAs:Cas9. The ribonucleoprotein (RNP) complex was incubated at room temperature for 10 minutes. 0.5–2 ×10^6 NK cells were collected, washed twice with PBS, pelleted, and resuspended in 20 μl of the RNP complex. NK cells were electroporated in Nucleocuvette strips using the X unit of a 4D-Nucleofector device (Lonza) using the EO-115 electroporation program. After electroporation, the cells were left to rest for 5 min at 37°C, then transferred into prepared flasks containing NK cell medium and cytokine, and cultured at 37°C.

### Quantification and statistical analysis

Data are presented as mean ± SD. An unpaired, nonparametric t-test (two-sided) was used to measure differences between groups. The difference between the survival curves was analyzed using the Kaplan-Meier method, and differences between groups were evaluated using the log-rank (Mantel-Cox) test. The time-series data were analyzed using a mixed-effects model and Šídák’s multiple-comparison test. Experimental sample sizes (n) and statistical analysis methods are included in the figure legends. Graphs were generated using the GraphPad Prism software (GraphPad, La Jolla, CA).

## Supplementary Material

Supplementary Files

This is a list of supplementary files associated with this preprint. Click to download.

- Supplementaryfiles.pdf

## Figures and Tables

**Figure 1:**
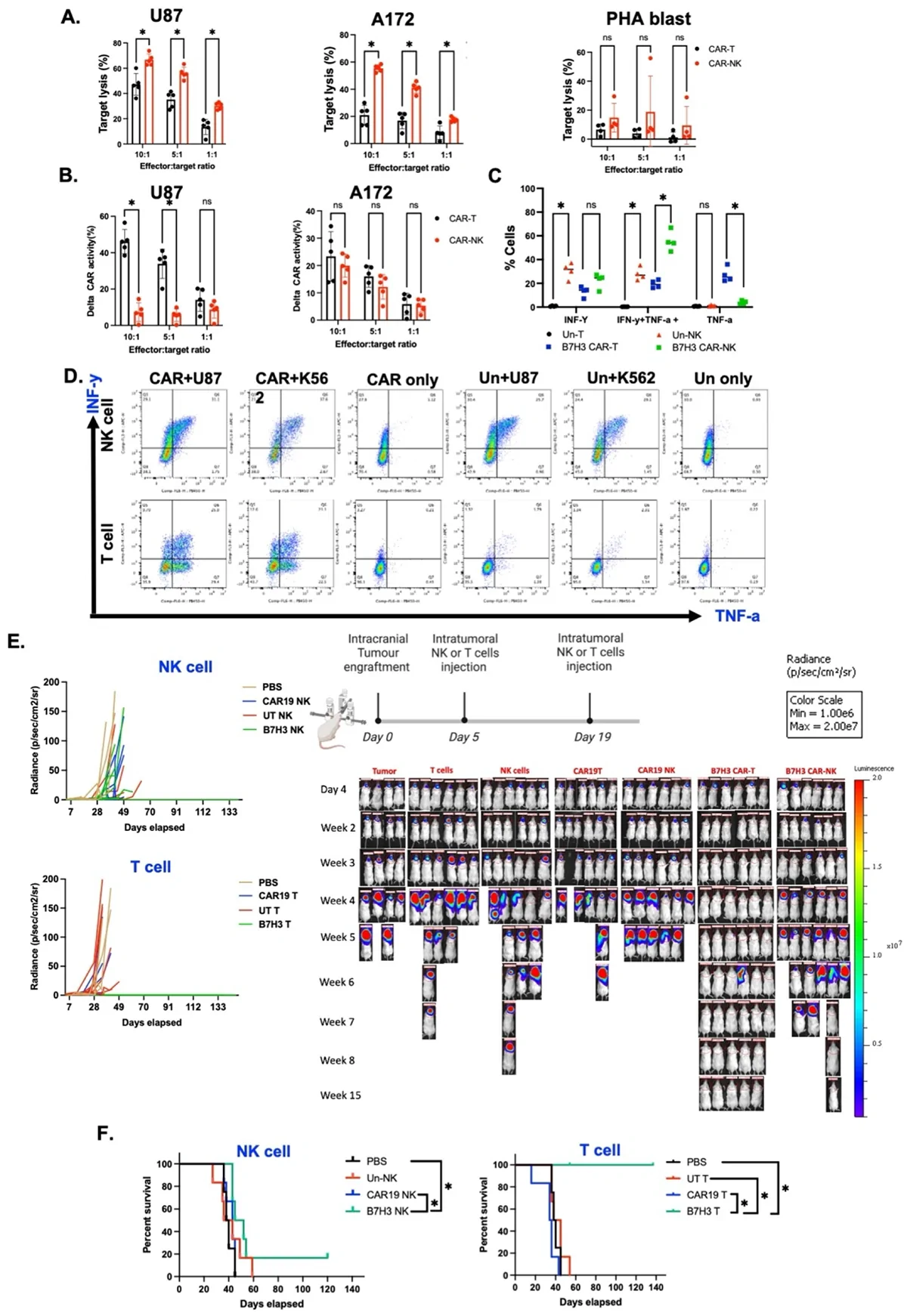
B7H3/CD28z NK and T Cells show potent antitumor activity, with NK cells outperforming in vitro and T cells in vivo. **(A)** Target cell lysis (%) by CAR-T and CAR-NK cells as measured by a 4-hour cytotoxicity assay using adult GBM cell lines U87 MG (n = 4), A172 (n = 4), and autologous PHA blast (n = 4 donors). **(B)** CAR-specific activity- the increase in NK or T cell-antigen-specific killing activity against U87 MG and A172 cells, as adjusted for the contribution of CAR expression. Data were analyzed by the Mann-Whitney test and presented as mean ± SD. **(C)**Antigen-specific activity as assessed by intracellular staining (ICS) for IFN-γ and TNF-α cytokines (n = 4 donors). **(D)** Representative flow cytometer data analysis for IFN-γ and TNF-α ICS from 4 individual healthy donors. Data were analyzed using the Mann-Whitney test and presented as mean ± SD. **(E)**Schema showing the in vivo experimental design (created using Biorender.com). Progression of luciferase-expressing U87 MG tumor was measured using bioluminescence imaging, and radiance was calculated using Living Image software. Radiance values for individual mice are shown. **(F)**Kaplan-Meier analysis of survival (n = 4–6 mice per group).

**Figure 2:**
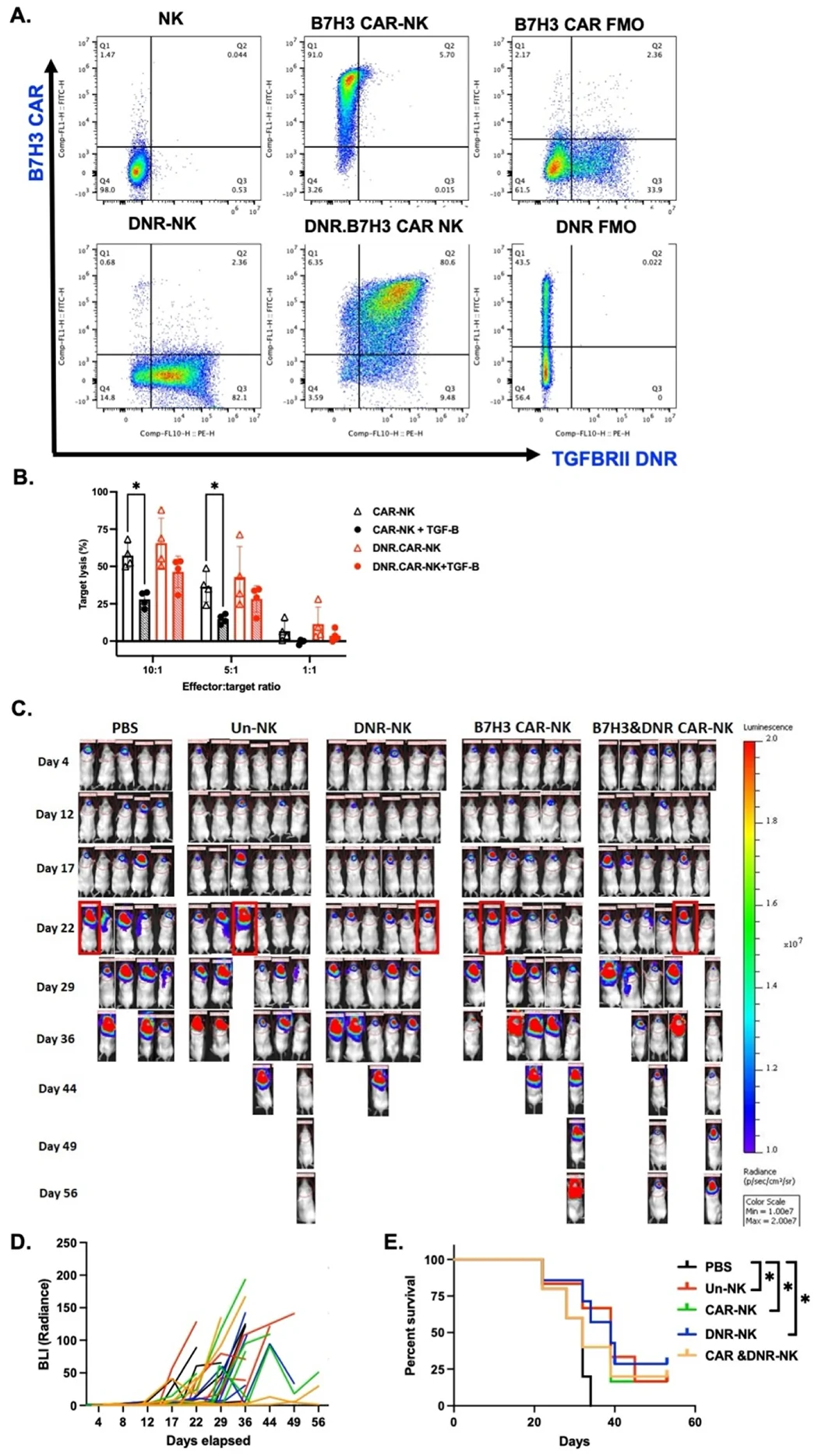
Overcoming TGF-β-mediated immunosuppression fails to enhance CAR-NK cells’ antitumor response in the NSG mouse model. (A) Representative flow cytometer data analysis for B7H3 CAR and TGFBRII DNR co-transduction in NK cells (n= 8 donors). (B) Lysis of target tumor cells (adult PDX cell line GC272) by CAR-NK cells as measured by a 4-hour cytotoxicity assay after treatment with exogenous TGF-β. (n=4 donors). Data were analyzed using the Mann-Whitney test and presented as mean ± SD. (C-D) Progression of luciferase-expressing U87 MG tumors was measured using bioluminescence imaging, and radiance was calculated using Living Image software. Radiance values for individual mice are shown. (E) Kaplan-Meier analysis of tumor-engrafted NSG mice treated with CAR-NK cells or control NK cells. Survival differences were analyzed using the log-rank (Mantel–Cox) test. (n = 4–6 mice per group).

**Figure 3:**
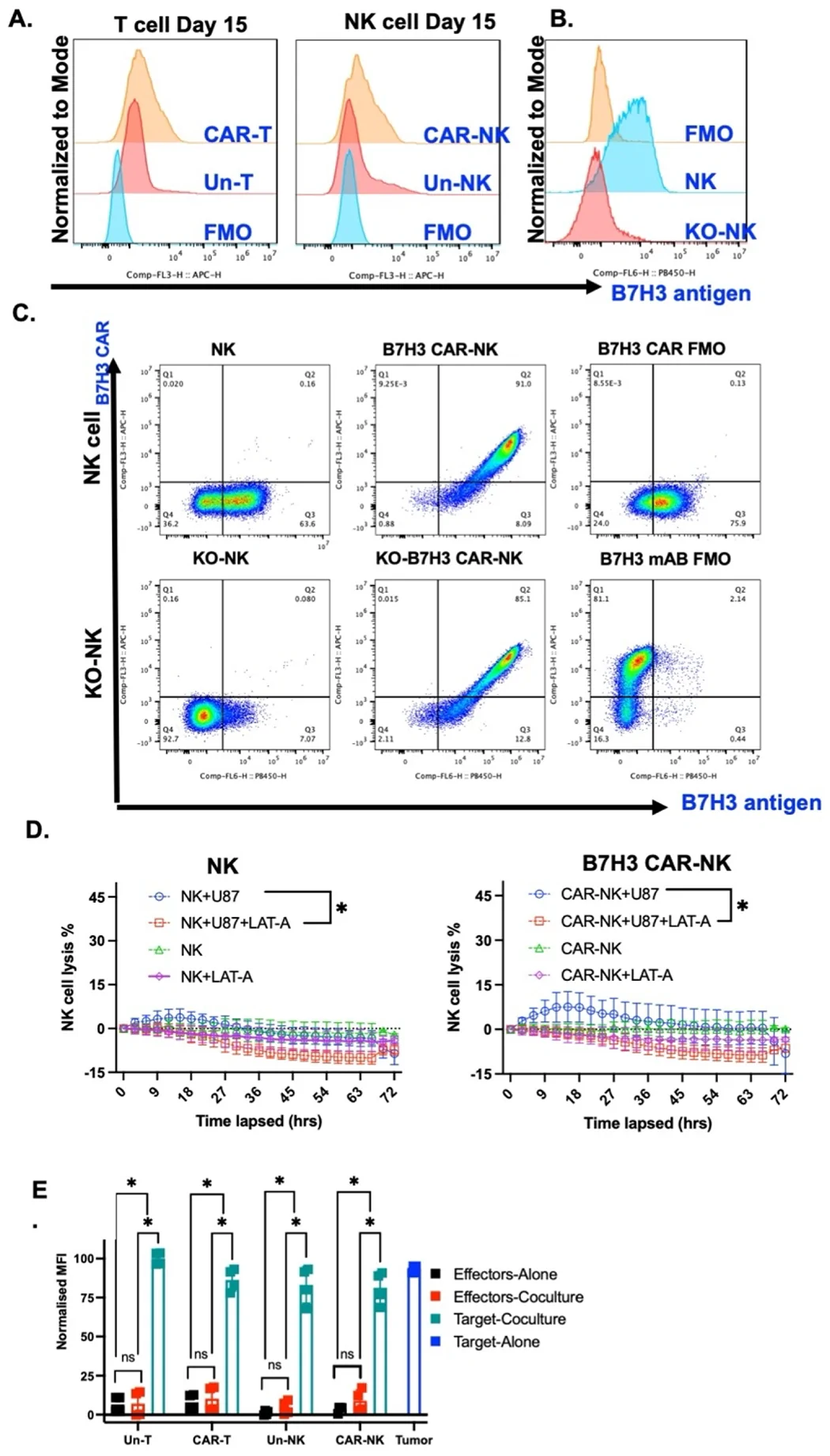
A high level of B7H3 persists on CAR-NK cells despite B7H3 knockout and is likely due to limited trogocytosis. **(A)** Median Fluorescence Intensity (MFI) of surface B7H3 in T and NK cells. Representative data from 4 individual healthy donors. **(B)** MFI of surface B7H3 expression in control or B7H3 knockout (KO) NK cells. Representative data from 4 individual healthy donors. **(C)** Representative flow cytometer data analysis for B7H3 antigen and B7H3 CAR expression in NK cells. (n = 4 donors). **(D)**Percentage of residual NK or CAR-NK cell lysis following coculture with the target at a 2:1 ratio in the presence and absence of LAT-A inhibitor. Data were analyzed using a mixed-effects model with Šídák’s multiple comparisons test and presented as mean ± SD. (n = 6 donors). **(E)** B7H3 MFI change on NK and tumor cells following coculture. Data were analyzed using the Mann-Whitney test, and presented as mean ± SD (n=4 donors).

**Figure 4:**
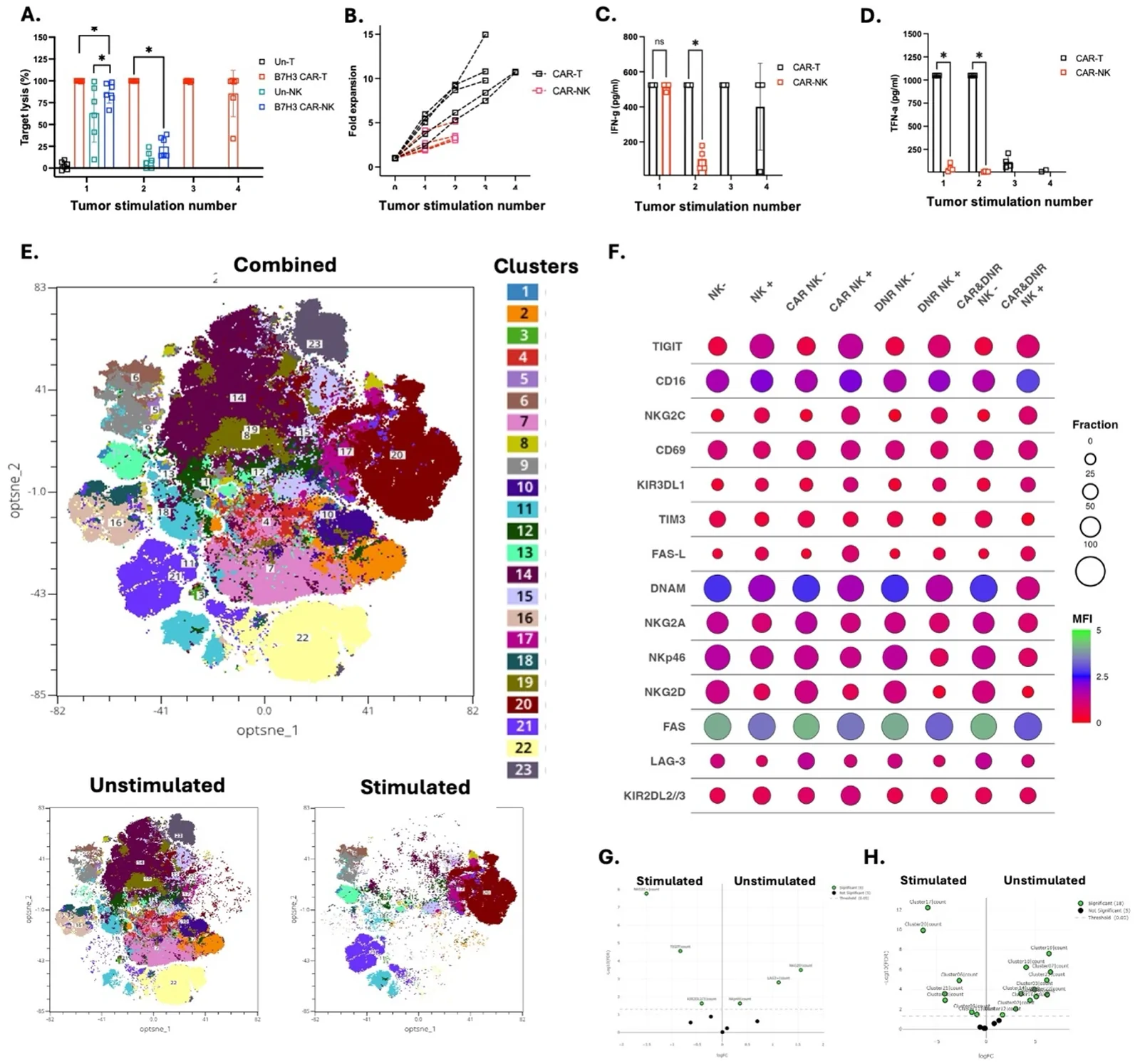
CAR-NK cells undergo senescence during repeated tumor challenges. **(A)** B7H3 CAR-transduced NK or control untransduced (Un)-NK or Un-T cells were cocultured with U87 MG tumor cells at a 2:1 ratio, with restimulation every 3–5 days using fresh tumor cells, until NK cells no longer killed or expanded, and for T cells, a maximum of 4 stimulations. Data were analyzed using the Mann-Whitney test and presented as mean ± SD. (n=4 donors). **(B)**Graphs show fold expansion of different NK and T-cell donors upon repetitive stimulations, each stimulation with fresh U87 MG tumor cells (n = 4). **(C)**IFN-γ (n = 4 donors) and **(D)** TNF-α (n = 4 donors) levels as detected by ELISA in the supernatants of cocultures after each stimulation during repeated tumor challenges. Data were analyzed using the Mann-Whitney test and presented as mean ± SD. **(E)** NK marker expression analysis by Aurora for transduced and untransduced NK cells (n = 3 donors), either unstimulated or after repeated tumor stimulations with U87 MG cells until they no longer killed and/or expanded. tSNE plots showing the cluster distribution. **(F)** Comparative heatmap showing the expression of NK cell markers in different conditions of NK cells. Color scale shows the normalized MFI expression level for each marker (green: higher expression; red: lower expression), and the size indicates the fraction (% expression) for each category. **(G)** Volcano plot showing NK cell markers significantly upregulated in stimulated vs. unstimulated NK cells. **(H)**Volcano plot showing clusters significantly upregulated in stimulated vs. unstimulated NK cells. Dashed lines indicate significance thresholds (|log_2_FC| ≥ 1 and p < 0.05). Green dots represent significantly higher expression levels.

**Figure 5:**
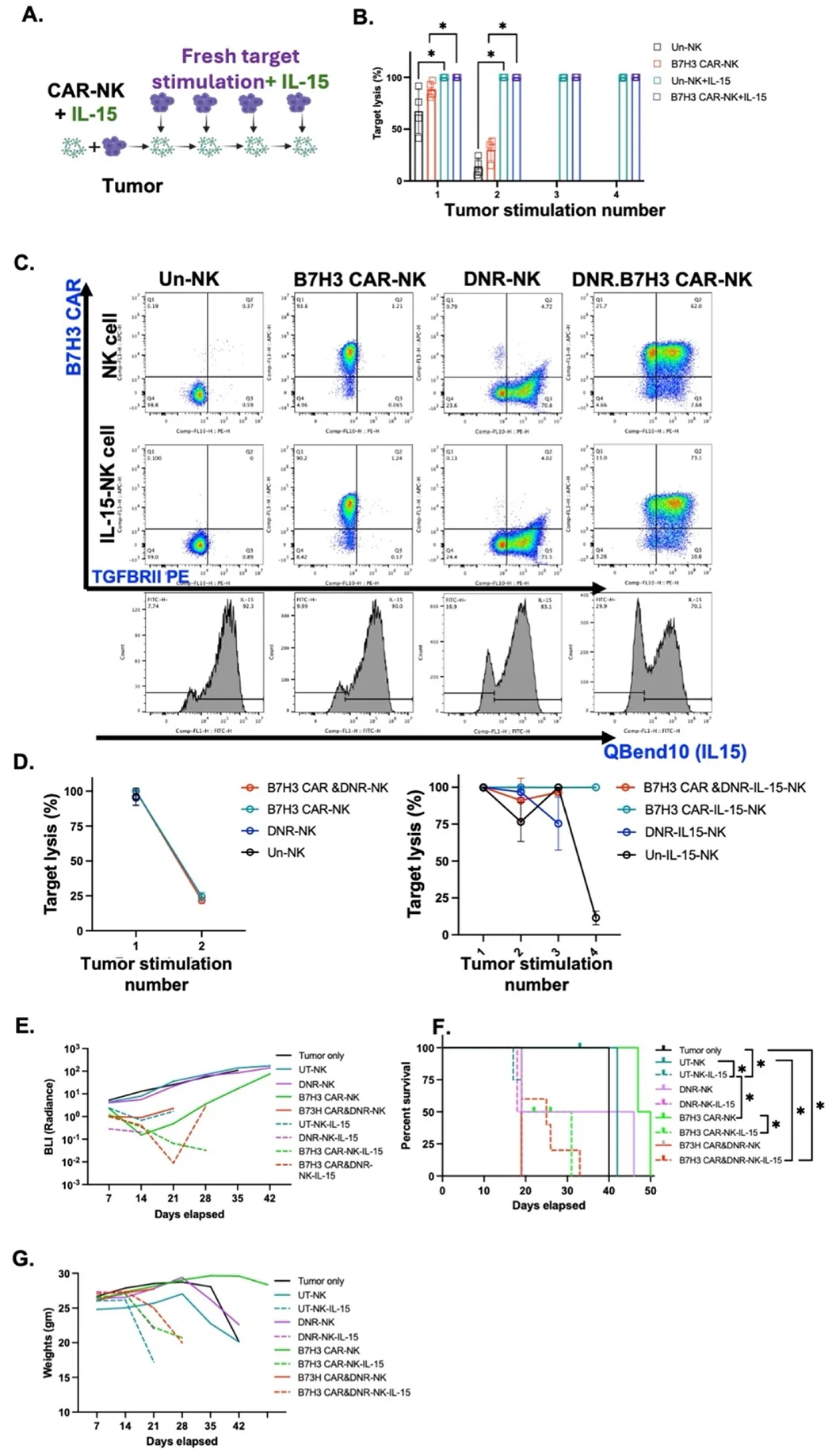
IL-15 rescues NK cells from senescence during repetitive tumor stimulations but does not improve survival. **(A)**Schematic of repeated tumor stimulations and gradual loss of killing ability by B7H3 CAR-NK cells and Un-NK cells, with and without exogenous IL-15 (created using Biorender.com). U87 MG target lysis by B7H3 CAR-NK and Un-NK cells with and without IL-15. Data are presented as mean ± SD. **(B)** Representative flow cytometer data of IL-15 expression in NK cells after transduction. (n = 2 donors). **(C)** Lysis of U87 MG target cells by control and IL-15-expressing NK cells. Data are presented as mean ± SD. **(D)**Progression of luciferase-expressing U87 MG-induced tumor was measured by bioluminescence imaging, and radiance was calculated using Living Image software. Radiance values for individual mice are shown. **(E)**Kaplan-Meier analysis of mice survival (n = 2–6 mice per group). **(F)**Changes in the body weight of mice over time across different NK cell treatment groups.

**Figure 6:**
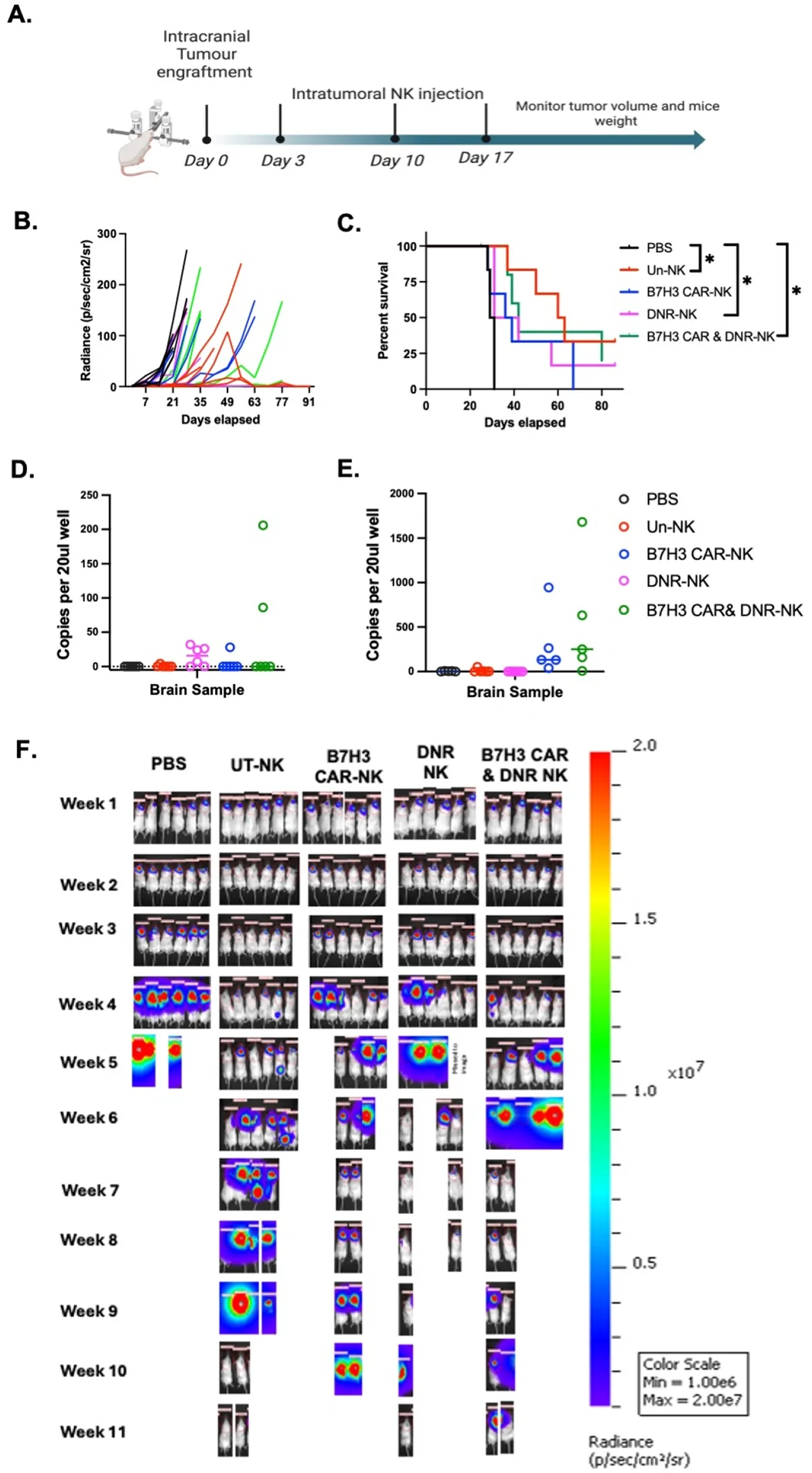
Early and frequent NK cell administration provides a survival advantage to mice. **(A)** Schema showing the in vivo experiment design (created using Biorender.com). NSG mice were injected intracranially with 2.5 × 10^5 tumor cells on day 1. NK cells: Un-NK, B7H3 CAR-NK, DNR-NK, and B7H3 CAR&DNR-NK (3 × 10^6 cells per injection) or PBS were administered intracranially at the same stereotactic coordinates 48 hours after tumor implantation, followed by two additional injections at 7-day intervals. **(B)** Tumor progression was monitored by bioluminescence imaging, and radiance was quantified using Living Image software (PerkinElmer). Radiance values for individual mice are shown. **(C)**Kaplan-Meier survival analysis of tumor-engrafted NSG mice treated with CAR-NK cells or control NK cells. Survival differences were analyzed using the log-rank (Mantel-Cox) test. (n = 6 mice per group). **(D)** CAR transgene copy number detected in the brain tissue by CD28ζ-ddPCR at the end of the study. Data represent n = 5–6 individual mice per treatment group. **(E)** DNR transgene copy number detected in the brain tissue by ddPCR at the end of the study. Data represent n = 5–6 individual mice per treatment group. **(F)**Representative bioluminescence images of tumor-bearing mice from each treatment group at the indicated time points.

## Data Availability

All data generated or analyzed during this study are included in this published article or its supplementary information files.
